# Enzymatic Activity and Its Relationship with Organic Matter Characterization and Ecotoxicity to *Aliivibrio fischeri* of Soil Samples Exposed to Tetrabutylphosphonium Bromide

**DOI:** 10.3390/s21051565

**Published:** 2021-02-24

**Authors:** Arkadiusz Telesiński, Barbara Pawłowska, Robert Biczak, Marek Śnieg, Jacek Wróbel, Dorota Dunikowska, Edward Meller

**Affiliations:** 1 Department of Bioengineering, Faculty of Environmental Management and Agriculture, West Pomeranian University of Technology in Szczecin, 17 Słowackiego St., 71-434 Szczecin, Poland; jacek.wrobel@zut.edu.pl (J.W.); dorota.dunikowska@zut.edu.pl (D.D.); 2 Department of Biochemistry, Biotechnology and Ecotoxicology, Faculty of Science and Technology, Jan Długosz University in Częstochowa, 13/15 Armii Krajowej Av, 42-200 Częstochowa, Poland; b.pawlowska@ujd.edu.pl (B.P.); r.biczak@ujd.edu.pl (R.B.); 3 Department of Agroengineering, Faculty of Environmental Management and Agriculture, West Pomeranian University of Technology in Szczecin, 3 Papieża Pawła VI, 71-459 Szczecin, Poland; marek.snieg@zut.edu.pl; 4 Department of Environmental Management, Faculty of Environmental Management and Agriculture, West Pomeranian University of Technology in Szczecin, 17 Słowackiego St., 71-434 Szczecin, Poland; edward.meller@zut.edu.pl

**Keywords:** enzyme activity index, FT-IR spectroscopy, ionic liquid, Microtox^®^, soil enzymes

## Abstract

This study aimed to determine the impact of tetrabutylphosphonium bromide [TBP][Br] on the soil environment through an experiment on loamy sand samples. The tested salt was added to soil samples at doses of 0 (control), 1, 10, 100, and 1000 mg kg^−1^ dry matter (DM). During the experiment, the activity of selected enzymes involved in carbon, phosphorus, and nitrogen cycles, characteristics of organic matter with Fourier-transform infrared (FT-IR) spectroscopy, and toxicity of soil samples in relation to *Aliivibrio fischeri* were determined at weekly intervals. The results showed that low doses of [TBP][Br] (1 and 10 mg kg^−1^ DM) did not have much influence on the analyzed parameters. However, the addition of higher doses of the salt into the soil samples (100 and 1000 mg kg^−1^ DM) resulted in a decrease in the activity of enzymes participating in the carbon and phosphorus cycle and affected the activation of those enzymes involved in the nitrogen cycle. This may be due to changes in aerobic conditions and in the qualitative and quantitative composition of soil microorganisms. It was also observed that the hydrophobicity of soil organic matter was increased. Moreover, the findings suggested that the soil samples containing the highest dose of [TBP][Br] (1000 mg kg^−1^ DM) can be characterized as acute environmental hazard based on their toxicity to *Aliivibrio fischeri* bacteria. The increased hydrophobicity and ecotoxicity of the soil samples exposed to the tested salt were also positively correlated with the activity of dehydrogenases, proteases, and nitrate reductase. Observed changes may indicate a disturbance of the soil ecochemical state caused by the presence of [TBP][Br].

## 1. Introduction

In recent years, increasing scientific attention has been paid to the biological and biochemical characteristics of soil, which are reported to be more sensitive to minor changes than the chemical or physical properties [1]. Among the indigenous biological components of soil, microorganisms play a key role in many important biochemical processes, such as the cycles of elements (carbon, nitrogen, phosphorus, and sulphur) and energy transfer taking place in the soil environment [2].

The basic source of microbial activity occurring in the soil is organic matter. Microorganisms are constantly involved in regulating the transformation of soil organic matter (SOM) [3]. Organic matter and microorganisms should not be considered as separate entities, but rather a united system constantly in close association and interactions with each other in soil environments. Interactions of these components have an enormous impact on terrestrial processes critical to environmental quality and ecosystem health [4]. Stress conditions caused by unfavorable anthropogenic effects may result in abnormal changes in microbial diversity or biologically active components of organic matter in the soil, including microbial biomass, enzymes, or various organic compounds, such as proteins or carbohydrates [5].

The activity of enzymes is one of the most useful parameters to assess the quality of the soil. The enzymes present in soil are mainly of microbial origin, but they are also secreted by plant roots and soil fauna [6]. Many authors have reported that enzyme activity is the most sensitive indicator of soil ecochemical status, due to the fact that enzymes participate in all microbiological reactions, including the cycles of soil nutrients, and react quickly to changes caused in soil by natural or anthropogenic factors [7,8,9].

One of the popular groups of chemicals currently used as substitutes for traditional organic solvents is ionic liquids (ILs). These compounds are non-volatile, non-flammable, and their “green” character is usually justified with their negligible vapor pressure. Therefore, they have attracted considerable interest as excellent alternatives to organic solvents to be used in homogeneous and biphasic processes [10]. Unlike traditional solvents, ILs are liquid salts that are entirely composed of ions. They usually consist of an organic cation and a much smaller inorganic or organic anion [11]. The positive charge of ILs is attributed to a nitrogen, phosphorus, or sulfur atom [12]. The low vapor pressure of these compounds has often been associated with their lack of toxic effects on the environment. However, it cannot be excluded that residual ILs are found in sewage and various elements of nature, both animate and inanimate [13]. In addition, products and wastes containing ILs may constitute a source of contamination. Pollution introduced directly into the water and soil environment as a result of various causes, such as failure of technological equipment and vehicle transportation, should also be considered [14].

One of the increasingly used ionic liquids is tetrabutylphosphonium bromide. It is a compound used in many chemical reactions especially in organic synthesis [15,16,17]. Moreover, it is relatively cheap, which also means that the number of possible applications of this compound may grow.

Currently, it is becoming extremely important to test as many chemical compounds as possible, including solvents, in order to determine their impact on various elements of the environment. It should be remembered that prevention is better than cure, so it makes sense to test chemicals before large-scale industrial use, and full scientific data helps to decide whether to use them or not at the research stage. This is much less costly than using untested chemicals in production, which can then generate enormous costs associated with removing them from the environment or eliminating their harmful effects, as has happened on more than one occasion in history. The properties of ILs, including their ability to form hydrogen bonds, can significantly influence their distribution rate, bioavailability, biodegradation potential, and accumulation in the soil [18]. Alkyl substituents present in the cation cause an affinity for hydrophobic soil components, which may significantly affect the structure of SOM [19].

SOM is the main component of soils, which influences the processes associated with the transformation of organic pollutants. The course of these transformation processes depends on soil and climatic factors as well as the properties of the compounds themselves [20]. Due to the division of contaminants between the solid and liquid phases of soils, sorption/desorption, sequestration, and formation of bound residues, compounds with hydrophobic properties are retained in soils. However, the effect of contaminants on microbiological processes can change the SOM structure, affecting the spatial arrangement of certain functional groups, such as carboxylic and hydroxylic groups [21], which are responsible for the chemical reactivity and sorption of SOM. Recently, Fourier-transform infrared (FT-IR) spectroscopy was used to characterize the SOM composition and its changes [22]. The FT-IR spectroscopy enables rapid characterization of the composition of SOM by analyzing functional groups such as carboxylic groups (C=O), which are responsible for cation exchange, or alkyl groups (C–H), which are responsible for wettability [23].

Commercial biological tests can also be used to study the impact of pollutants on the soil ecosystem [24]. One of the most commonly used biological tests to assess the ecotoxicity of contaminated soil and soil-like materials is the Microtox^®^ assay which is carried out using the bioluminescent bacteria *Aliivibrio fischeri* [25]. Initially, this test was developed for analyzing contaminated water and wastewater, and later it was modified for evaluating sediment and soil in the direct contact test [26]. *Aliivibrio fischeri* are nonpathogenic, marine bacteria, which exhibit bioluminescence as a part of their natural metabolism. Under the influence of contact with contaminants, the respiratory process of the bacteria is disturbed, which reduces the light yield. This change in bioluminescence can be used to calculate the percentage inhibition of *A. fischeri*, which is directly related to toxicity [27].

One of the most frequently cultivated cereals in Poland is spring barley. It has a poorly developed root system (its roots develop the shallowest of all cereals) and is characterized by a short vegetation period, which makes it quite demanding in terms of soil requirements. Therefore, the presence of contaminants, including ionic liquids, in the soil can affect its growth and development [28]. The results of [TBP][Br] phytotoxicity for spring barley are described in another article [29]. Moreover, spring barley is indicated in Polish norm PN-EN ISO 11269-2 [30] as a recommended species for xenobiotic toxicity testing.

The aim of this study was to determine the effect of the IL—tetrabutylphosphonium bromide [TBP][Br] on the activity of enzymes involved in the cycles of carbon, nitrogen, characteristics of SOM, and ecotoxicity of soil.

## 2. Materials and Methods

### 2.1. Chemicals and Equipment

[TBP][Br] and all the substrates used for the assay of enzyme activity and the reagents used in the analyses were purchased from Sigma-Aldrich Chemical Co. (Poznań, Poland). Spectrophotometer UV-1800 (Shimadzu, Kyoto, Japan) was used for taking spectrophotometric measurements, Nicolet iS5 Mid Infrared FT-IR spectrometer (Termo Fisher Scientific, Warsaw, Poland), and Microtox^®^ LX equipment (Modern Water, London, UK) was used for ecotoxicity analysis.

Deionized water (HLP Smart 2000 demineralizer; Hydrolab, Straszyn, Poland) with an average specific conductivity of 0.15 μS cm^−1^ and a surface tension value of 72.3 mN m^−1^ at 25 °C was used for preparing the test solutions.

### 2.2. Experimental Design

The experiment was carried out according to Polish norm PN-EN ISO 11269-2 [30] and OECD/OCDE 208 guide [31] in the vegetation hall of the Department of Biochemistry, Biotechnology, and Ecotoxicology Jan Długosz University in Częstochowa. Loamy sand samples with an organic carbon (C_org_) content of 8.51 g kg^−1^ and a pH (in 1 mol dm^−3^ KCl solution) of 5.89 were used in the experiment. The samples collected in field from the depth 0–20 cm were dried to air-dry condition and sifted through a 2-mm mesh sieve. Soil samples prepared in this way were brought to 70% of maximum water holding capacity and incubated at 20 °C for 5 days. After this time [TBP][Br] was added to them at doses of 0 (control), 1, 10, 100, and 1000 mg kg^−1^ dry matter (DM). Four pots with a diameter of 90 mm, for each dose of IL, were filled with the soil samples prepared in this way (250 g). Then, 20 seeds of spring barley (*Hordeum vulgare* L.) were sown in each vase. During the experiment, the vases were illuminated with a sodium lamp at a radiation intensity of 170 μmol m^−2^ s^−1^ at the substrate level. Photoperiod was established at 16-h day and 8-h night. Soil moisture was maintained, and water losses were made up every two days by soil weight control using the scales. On days 1, 7, 14, and 28, the activity of enzymes involved in the C, N, and P cycle, infrared (IR) spectra absorption, and toxicity to *A. fischeri* were determined in soil samples. Soil samples were taken from different depths of each pot at each measurement term. A summary sample was then created, and it was used to determine soil enzyme activities, FT-IR analysis, and ecotoxicity assays. Analyses of all determined parameters were therefore performed in four replicates.

### 2.3. Determination of Soil Enzyme Activity

The activity of the following enzymes which are involved in the cycles was determined spectrophotometrically: phosphorus cycle—alkaline phosphomonoesterase (ALP; EC 3.1.3.1), acid phosphomonoesterase (ACP; EC 3.1.3.2), phosphodiesterase (PD; EC 3.1.4.1), and phosphotriesterase (PT; EC 3.1.8.1); nitrogen cycle—urease (URE; EC 3.5.1.5), nitrate reductase (NR; EC 1.7.1.1), proteases (PROT; EC 3.4.21), and arginine deaminase (ArgD; EC 3.5.3.6); and carbon cycle—dehydrogenases (DHA; EC 1.1.1), lipase (LIP; EC 3.1.1.3), and β-glucosidase (GLU; EC 3.2.1.21). Information on the methods of determination of these enzymes is presented in Table 1. In addition, the activity of catalase (CAT; EC 1.11.1.6) was determined by manganometry according to the method of Johnson and Temple [32], using hydrogen peroxide as a substrate.

### 2.4. Fourier-Transform Infrared (FT-IR) Spectroscopy

**Figure 1:**
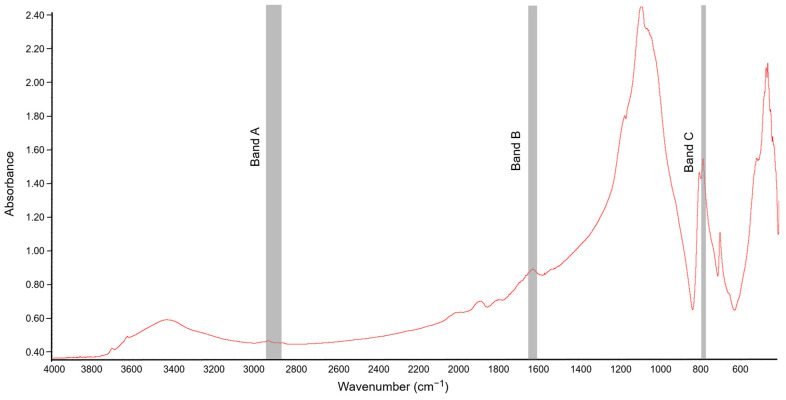
Fourier-transform infrared (FT-IR) spectrum of soil not exposed to tetrabutylphosphonium bromide [TBP][Br]; Band A, Band B, and Band C associated with absorbance of C–H bonds, hydrophilic components, and quartz, respectively.

FT-IR spectra of the soil samples were obtained using the potassium bromide (KBr) technique described by Celi et al. [43]. For obtaining the IR absorption spectra of the soil samples, lozenges were made by mixing 1 mg of soil with 200 mg KBr (spectroscopic grade). The prepared mixture was ground in an agate mortar. Before the preparation of lozenges, both KBr and soil samples were roasted for 24 h at 105 °C [21] to limit the absorption of moisture from the air, which could affect the interpretation of the spectrum. The spectroscopic analyses were carried out in the mid-IR range with a spectral area from 4000 to 400 cm^−1^ at a resolution of 1 cm^−1^. Thirty-two scans were recorded for each sample, averaged, and corrected against the ambient air (H_2_O and CO_2_) as background [23]. An example spectrum obtained for soil sample not exposed to [TBP][Br] is shown in Figure 1.

To analyze the chemical nature of SOM, three absorption bands were selected and the peak height at their maximum absorption was determined. The “A” band represented the aliphatic fraction of SOM (2947–2858 cm^−1^), the “B” band represented the hydrophilic part of SOM (1647–1633 cm^−1^), and the “C” band was associated with the Si–O bond in quartz (798–779 cm^−1^) [21]. The peak height of the “A” band and that of the “B” band were normalized using the peak height of the “C” band, which was considered as an internal reference point. Quartz is present in all soils, and so its absorption band is characteristic and its signal is not affected by other minerals [44]. In this study, the ratio of the peak heights of the A/C and B/C bands was determined to assess the contribution of the aliphatic and hydrophilic components of SOM, respectively. In turn, the ratio of the peak heights of the A/B bands was used to determine the ratio of the relative abundance of hydrophobic and hydrophilic functional groups [45,46].

### 2.5. Microtox^®^ Assay

The ecotoxicity of soil samples containing [TBP][Br] was determined using the incomplete acute solid-phase test with *A. fischeri* bioluminescent bacteria. Briefly, 7 g of the summary soil sample was mixed for 10 min with 35 cm^3^ of 2% NaCl solution. Then, 1.5 cm^3^ of soil suspension was taken as the diluent, which corresponded to 0.3 g of the soil sample, and was added to the solution containing the bioluminescent bacteria. This allowed the bacteria to come into direct contact with the solid sample in the form of solids in the aqueous suspension, thus enabling the determination of the toxicity of not only water-soluble substances but also compounds such as lipophilic ones [47]. The greater the reduction in light emitted by the bacteria, the greater the toxicity of the sample. To measure the luminescence, photometer of Microtox^®^ LX was used. It was designed specifically for use with Modern Water’s bioluminescent bacteria. For the purpose of the study, no subsequent sample dilutions were performed, and the results were expressed as a percentage of soil toxicity after 30 min of contact with *A. fischeri* bacteria.

### 2.6. Data Analysis

The obtained results were statistically analyzed by analysis of variance. The dose of ILs and the time of exposition were main experimental factors. One-way analysis of variance was used for comparison of results of soil enzymes activity. It was performed independently for each experimental factor. Whereas for comparison of results of enzyme activity index (*EAI*), FT-IR, and ecotoxicity two-ways analysis of variance was used. Post hoc Tukey’s honestly significant difference (HSD) test at *p* = 0.05 was used to determine the significance of changes. To assess the effect of [TBP][Br] on the activity of the analyzed enzymes, relative activity (*RA*) was calculated according to the formula:

(1) $$RA=\frac{A_{IL}}{A_{C}}$$

where *A_IL_* is the enzyme activity in the soil exposed to [TBP][Br] and *A_C_* is the enzyme activity in the control soil. Additionally, to determine the total effect of [TBP][Br] on the activity of particular groups of enzymes participating in the carbon, phosphorus, and nitrogen cycle, the consolidated enzyme activity index (*EAI*) was calculated according to the formula given by Różyło and Bohacz [48]:

(2) $$EAI=\frac{1}{n}\overset{n}{\underset{i=1}{\sum }}RA\left(n\right)$$

*EAI* was calculated separately for the enzymes involved in the carbon, phosphorus, and nitrogen cycle.

To determine the magnitude of the influence of the experimental factors on the examined biochemical parameters and SOM characteristics, an η^2^ analysis was carried out, which describes the ratio of the variance of the dependent variable explained (in a purely correlative sense) by an independent variable (predictor) [49].

To assess the relationship between the activity of the enzymes determined and the SOM characteristics and ecotoxicity of the soil samples, Pearson’s linear correlation coefficients were also calculated (*p* = 0.05), and an exploratory factor analysis was performed. Statistica 13.2 software was used for the statistical analyses.

## 3. Results and Discussion

### 3.1. Enzyme Activity in the Soil Exposed to Tetrabutylphosphonium Bromide [TBP][Br]

The activity of the enzymes in the soil that was not exposed to [TBP][Br] remained at a similar level during the experiment (Table 2). This is in line with the results of our earlier study, in which no significant changes were found in the activity of enzymes over time in uncontaminated soil [7,50,51,52,53,54,55].

**Figure 2:**
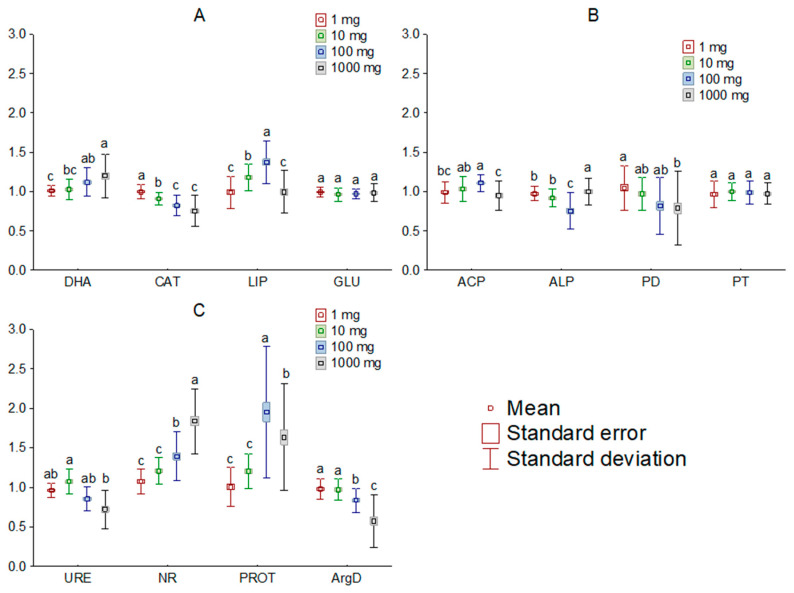
Mean relative activity of the enzymes involved in carbon (**A**), phosphorus (**B**), and nitrogen (**C**) cycle in the soil exposed to tetrabutylphosphonium bromide [TBP][Br] in pot experiment with spring barley, depending on the salt doses: values denoted by the same letters for each enzyme do not differ significantly on the level of *p* = 0.05 (Tukey HSD test), DHA—dehydrogenases, CAT—catalase, LIP—lipase, GLU—β-glucosidase, ACP—acid phosphomonoesterase, ALP—alkaline phosphomonoesterase, PD—phosphodiesterase, PT—phosphotriesterase, URE—urease, NR—nitrate reductase, PROT—proteases, ArgD—arginine deaminase.

**Figure 3:**
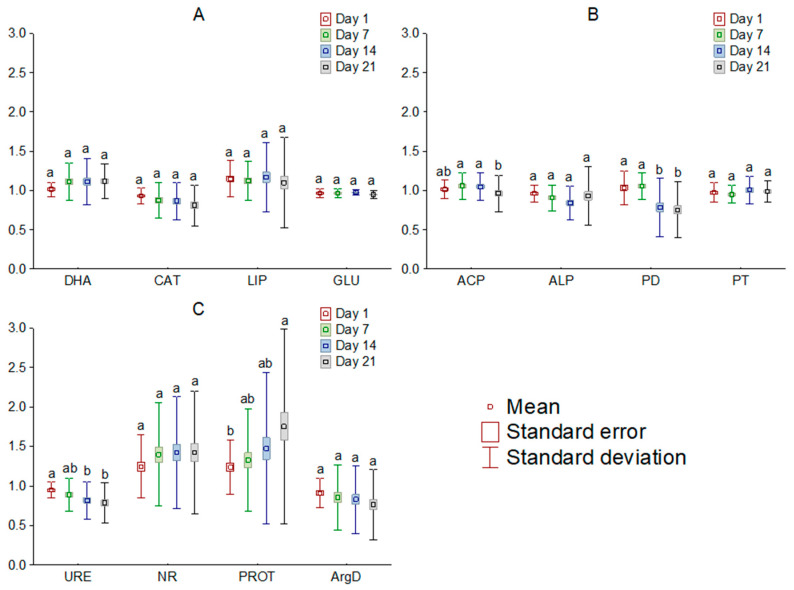
Mean relative activity of the enzymes involved in carbon (**A**), phosphorus (**B**), and nitrogen (**C**) cycle in the soil exposed to tetrabutylphosphonium bromide [TBP][Br] in pot experiment with spring barley, depending on the day of the experiment: values denoted by the same letters for each enzyme do not differ significantly on the level of *p* = 0.05 (Tukey HSD test), DHA—dehydrogenases, CAT—catalase, LIP—lipase, GLU—β-glucosidase, ACP—acid phosphomonoesterase, ALP—alkaline phosphomonoesterase, PD—phosphodiesterase, PT—phosphotriesterase, URE—urease, NR—nitrate reductase, PROT—proteases, ArgD—arginine deaminase.

The addition of [TBP][Br] to the soil influenced the activity of most of the analyzed soil enzymes. Comparing the observed changes, based on the dose of IL, it was found that doses of 1 and 10 mg kg^−1^ DM, in most cases, had little effect on the activity of the determined enzymes, whereas the effect of higher doses tended to significantly affect soil biochemical parameters. Among the enzymes involved in carbon, phosphorus, and nitrogen cycling, the greatest changes occurred in LIP, ALP, and PROT, respectively (Figure 2). On the other hand, a comparison of changes by measurement date showed significant changes in activity only for PD, URE and PROT (Figure 3). Moreover, the greatest influence of [TBP][Br] was found for the activity of enzymes involved in the nitrogen cycle, especially NR and PROT. The increase in PROT may be due to the death and lysis of microbial cells under the influence of [TBP][Br] and the release of proteins from them, which are degraded by PROT [56]. In contrast, the stimulation of NR may be due to the disruption of air relations in the soil with [TBP][Br], as the reduction of NO3 to NO2 occurs under anaerobic conditions [57]. The η^2^ analysis showed that the activity of DHA, CAT, LIP, ACP, ALP, PD, URE, NR, PROT, and ArgD was most strongly influenced by the dose of [TBP][Br], while that of GLU and PT was most strongly influenced by the time of measurement (Table 3). The obtained results confirm the results of research conducted on other ILs which differ in both cation and anion. Most of the available studies concern the effect of imidazole ILs on the activity of soil enzymes, in which, depending on the cation structure and anion type, a decrease in the activity of DHA, ALP [58], URE [59], and peroxidases [60], as well as stimulation of the activity of GLU or ACP [59], was found. Some studies also showed the effect of quaternary ammonium salts (QAS) and other ILs on the activity of soil enzymes. These demonstrated, among others, a decrease in the activity of DHA under the influence of QAS with iodine anion [61] or the activity of o-diphenol oxidase under the influence of QAS with hexafluorophosphate anion [62]. These effects were observed to increase both with the increase in the dose of ILs and the level of elongation of alkyl substituent in the cation [58,59,60,61,62]. The disruption of soil enzyme activity is due to the high antimicrobial potential of ILs. Negative effects of various ILs have been demonstrated against many fungal and bacterial species, for example: *Escherichia coli*, *Staphylococcus aureus*, *Bacillus subtilis*, *Pseudomonas fluorescens*, and *Saccharomyces cerevisiae* [63,64,65,66]. Several authors also report similar microbial toxicity of ILs and traditional organic solvents [67,68].

### 3.2. Enzyme Activity Index (EAI) of the Soil Exposed to Tetrabutylphosphonium Bromide [TBP][Br]

**Figure 4:**
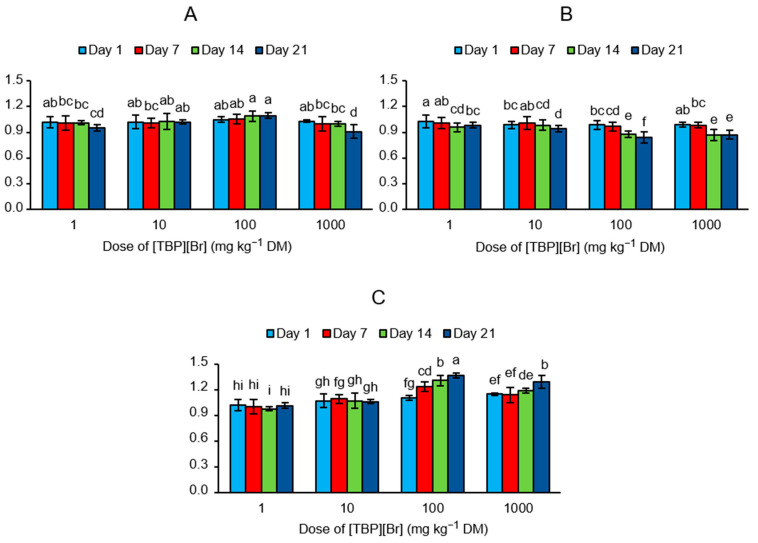
Values of enzyme activity indices (*EAI*) determined for the soil exposed to tetrabutylphosphonium bromide [TBP][Br] in pot experiment with spring barley: (**A**) carbon cycle, (**B**) phosphorus cycle, (**C**) nitrogen cycle; data are presented as mean ± SD; values denoted by the same letters for each enzyme do not differ significantly on the level of *p* = 0.05 (Tukey HSD test).

The obtained values of *EAI* showed that the addition of [TBP][Br] at a dose of 1 and 10 mg kg^−1^ DM had only a small effect on the activity of all groups of the analyzed enzymes, whereas at higher doses of the IL, the greatest influence was recorded on the last measurement date (Figure 4). After the addition of the tested IL at a dose of 100 mg kg^−1^ DM, activation of the enzymes participating in the carbon cycle (*EAI* = 1.10) and nitrogen cycle (*EAI* = 1.36) and inhibition of the enzymes participating in the phosphorus cycle (*EAI* = 0.84) were observed on day 21. However, with the addition of 1000 mg kg^−1^ DM of IL, inhibition of the enzymes participating in the carbon cycle (*EAI* = 0.91) and phosphorus cycle (*EAI* = 0.87) and activation of the enzymes participating in the nitrogen cycle (*EAI* = 1.29) were observed on the last day of incubation.

It has been shown that the changes in the soil enzyme activity under the influence of ILs result from disturbed microbial biodiversity [58]. For instance, Zhang et al. [69] demonstrated a decrease in the number of all groups of microorganisms (bacteria, actinomycetes, and fungi) under the influence of 1-octyl-3-methylimidazolium hexafluorophosphate. Similar results were observed by Cheng et al. [59] for the use of imidazole-based ILs with bromide anion and Sydow et al. [70] for phosphonium-based ILs. Moreover, some authors have reported that exposure to ILs may reduce the number of AOB-amoA and AOA-amoA genes in the soil [59,71], which directly or indirectly affects the oxidation of ammonia by soil microorganisms, and thus reduces the expression of related genes. The inhibition of AOB and AOA can also reduce the ability of the soil to nitrify and hence affect the nitrogen cycle in the soil [59]. This may explain the greatest changes observed in our study in the activity of the enzymes involved in the nitrogen cycle.

### 3.3. Characterization of Soil Organic Matter by FT-IR Spectroscopy in the Soil Exposed to Tetrabutylphosphonium Bromide [TBP][Br]

The addition of [TBP][Br] to the soil at doses of 1 and 10 mg kg^−1^ DM did not cause any significant changes in the values of A/C and B/C ratios, as compared to the control soil, during the experiment. On the other hand, after the application of the tested IL at doses of 100 and 1000 mg kg^−1^ DM, the values of A/C ratio were significantly increased from day 7. However, the values of B/C ratio were found to decrease significantly from day 7 only for the soil samples treated with IL at a dose of 1000 mg kg^−1^ DM. For the soil samples exposed to IL at the dose of 100 mg kg^−1^ DM, significantly lower values of the B/C ratio were recorded only on day 21. These results indicate an increase in the share of aliphatic compounds and a decrease in the share of hydrophilic compounds in the SOM under the influence of higher doses of [TBP][Br]. Additionally, the calculated values of the A/B ratio, which were higher for the soil exposed to IL at doses of 100 and 1000 mg kg^−1^ DM compared to the control soil on day 7, and at doses of 10, 100, and 1000 mg kg^−1^ DM on days 14 and 21, indicate the advantage of hydrophobic substances present in the soil containing the tested IL (Table 4).

The presence of hydrophilic and hydrophobic functional groups in the SOM is determined by the spatial structure of carbon chains. These functional groups are responsible for the chemical reactivity and adsorption properties of the organic matter [21]. They also determine the ability to form hydrophobic and hydrogen-related interactions or the ability to carry out cation exchange with the absorbed substance and other soil components such as clay minerals [72].

Sorption plays an important role in the toxicity of ILs in the soil [73]. The results from available studies show that the cations present in ILs with long-chain hydrophobic substitutes can easily adsorb on different types of soil, and become persistent contaminants in the environment [74,75,76]. On the other hand, compounds containing short alkyl substituents with additional polar functional groups can be easily transported into the soil, and thus pose a risk of groundwater contamination [18,77]. It should be noted that lower toxicity of ILs has been observed in the soil with a high content of organic carbon [60,78,79].

Moreover, the increased hydrophobicity of SOM may cause a longer persistence of [TBP][Br] in soil. This is due to the fact that hydrophobic interaction of ILs with SOM is the main sorption mechanism of these compounds in soil [80,81,82].

The η^2^ analysis showed that the values of A/C, B/C, and A/B ratios were influenced by the dose of [TBP][Br] (Table 5). However, Zhou et al. [83] did not find any influence of imidazole ILs with nitrate (V) anion on the cation exchange capacity of the soil, regardless of the dose of the substance or the exposure time.

### 3.4. Ecotoxicity of the Tetrabutylphosphonium Bromide [TBP][Br]-Exposed Soil to A. fischeri

**Figure 5:**
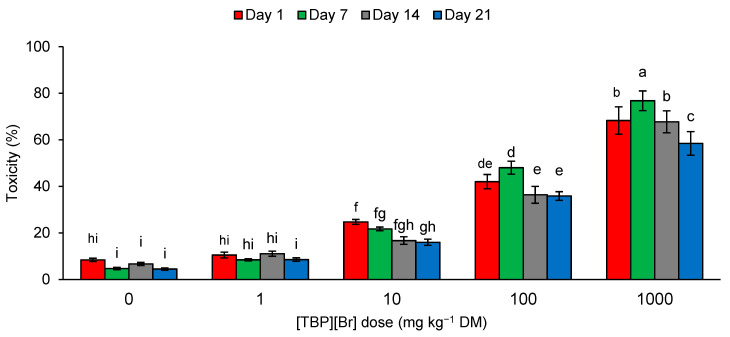
Ecotoxicity of tetrabutylphosphonium bromide [TBP][Br]-exposed soil to *A. fischeri* in pot experiment with spring barley; data are presented as mean ± SD; values denoted by the same letters do not differ significantly at the level of *p* = 0.05 (Tukey HSD test).

During the experiment, the toxicity of the control soil samples and samples containing [TBP][Br] at a dose of 1 mg kg^−1^ DM ranged from 4.50 to 11.09%. An increase in the dose of the tested IL caused an increase in the toxicity of the soil samples. The toxicity observed for the dose of 10 mg kg^−1^ DM was 16.00–24.74%, for 100 mg kg^−1^ DM was 35.85–48.00%, and for 1000 mg kg^−1^ DM was 58.43–76.78% (Figure 5). According to the scale given by Persoone et al. [84], soil samples containing [TBP][Br] at a dose of 100 mg kg^−1^ DM are characterized by low ecotoxicological risk, and those containing the salt at a dose of 1000 mg kg^−1^ DM by acute ecotoxicological risk. In other cases, the samples can be classified as nontoxic. According to the toxicity assessment procedure for environmental samples proposed by these authors, toxicity in the unit of percentage is used to select toxic samples. The percentage of toxic effect is compared with the control, assuming that an effect below 20% means that the sample is nontoxic. The application of the system of toxicity classification developed by Persoone et al. [84] is often used to assess the ecotoxicity of samples contaminated with various xenobiotics [25,85,86,87,88,89]. A number of reports on the use of *A. fischeri* bacteria and Microtox^®^ in the assessment of the ecotoxicity of ILs can be found in the literature. However, due to the large variation in the structure of the tested compounds, the obtained results indicate different degrees of toxicity [65,90,91,92,93].

### 3.5. Relationships Between Enzyme Activity and Organic Matter Characterization and Ecotoxicity to A. fischeri of the Soil Samples Exposed to Tetrabutylphosphonium Bromide [TBP][Br]

The calculated coefficients showed that if a relationship existed between the activity of a given enzyme and the values of A/C ratio in the soil samples containing [TBP][Br], a significant relationship can also be found between the values of A/B ratio and ecotoxicity. Such positive correlations were noted for DHA among the enzymes involved in the carbon cycle and for NR and PROT among the enzymes involved in the nitrogen cycle. These enzymes were also negatively correlated with the values of B/C ratio. However, a significant negative correlation was found between the values of A/C ratio, A/B ratio, and ecotoxicity and the activity of CAT among the enzymes involved in the carbon cycle, PD among the enzymes involved in the phosphorus cycle, and URE and ArgD among the enzymes involved in the nitrogen cycle (Table 6). These enzymes were also positively correlated with the values of B/C ratio. Therefore, it can be concluded that the activity of DHA, NR, and PROT depends to a large extent on the hydrophobic properties of the soil and that of CAT, PD, URE, and ArgD depends on the hydrophilic properties.

Many authors have shown a positive correlation between SOM content and the activity of various enzymes taking [94,95,96,97,98]. However, in addition to SOM content, the quality and composition of SOM also have a significant influence on enzyme activity. The interaction of enzymes with SOM components has been the subject of many studies [99]. As reported Olagokeet al. [100] there is a constant amount of free enzymes in the soil, but the greater part of the enzymes are permanently bound to SOM components. Enzymes have been shown to vary in their binding to different functional groups of SOM [101]. Associated enzymes are protected from diffusion into the soil and their activity becomes less sensitive to variations in temperature, moisture, and pH. Changes in the distribution of hydrophilic and hydrophobic properties of SOM cause the release of enzymes into the soil solution increasing their susceptibility to proteolysis [101].

**Figure 6:**
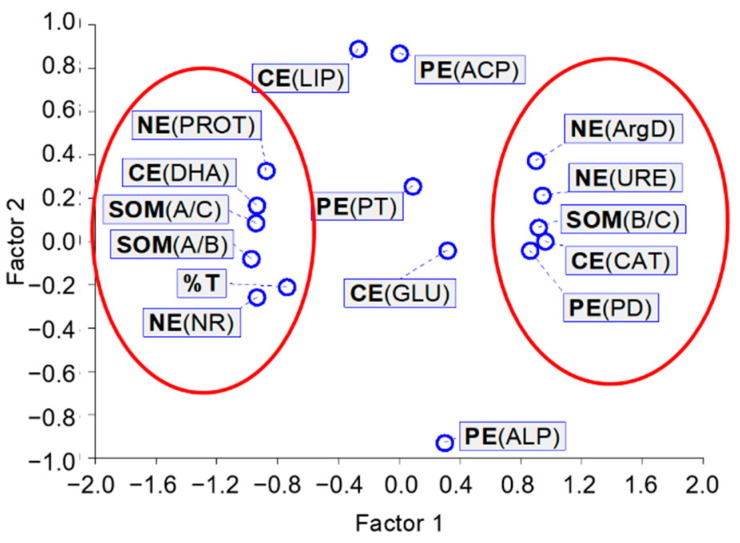
Characteristics of the exploratory factor analysis; CE—enzymes involved in carbon cycle: DHA—dehydrogenases, CAT—catalase, LIP—lipase, GLU—β-glucosidase; PE—enzymes involved in phosphorus cycle: ACP—acid phosphomonoesterase, ALP—alkaline phosphomonoesterase, PD—phosphodiesterase, PT—phosphotriesterase; NE—enzymes involved in nitrogen cycle: URE—urease, NR—nitrate reductase, PROT—proteases, ArgD—arginine deaminase; SOM—soil organic matter parameters: A/C contribution of aliphatic components, B/C contribution of hydrophilic components, A/B abundance of hydrophobic and hydrophilic groups; %T percentage toxicity to *A. fischeri*.

The results obtained for linear correlations were confirmed by the exploratory factor analysis, which clearly showed the grouping in one neighborhood, with a value of about −1 for factor 1, DHA, PROT, NR, A/C, A/B, and %T, and in the other neighborhood, with a value of about 1 for factor 1, CAT, PD, URE, ArgD, and B/C (Figure 6). It is interesting to note the close position next to each other of DHA—catalyzing the oxidation of organic compounds, NR—indicating the formation of anaerobic conditions, PROT—degrading proteins, released into the soil, and contribution of aliphatic SOM components and ecotoxicity for *A. fischeri*. In our opinion, this may indicate a disturbance of the soil ecochemical state caused by the presence of [TBP][Br].

## 4. Conclusions

ILs are compounds that have been considered environmentally friendly for many years. However, current reports show their negative impact on various elements of ecosystems. The results of the present study also indicate the adverse effects of [TBP][Br] on the soil environment. Although low doses of this salt (1 and 10 mg kg^−1^ DM) did not show much influence on the parameters determined, its addition at higher doses (100 and 1000 mg kg^−1^ DM) to the soil samples resulted in a decrease in the activity of enzymes involved in the carbon and phosphorus cycle and affected the activation of those enzymes participating in the nitrogen cycle, which is very well illustrated by the *EAI* values. The added salt also increased the hydrophobicity of SOM. Thus, the soil samples containing the highest dose of [TBP][Br] (1000 mg kg^−1^ DM) can be characterized as acute environmental hazard based on their toxicity to *A. fischeri* bacteria. Additionally, the increased hydrophobicity and ecotoxicity of the soil exposed to the tested salt were positively correlated with the activity of DHA, PROT, and NR. The use of [TBP][Br] can be beneficial in industrial terms. However, on the basis of the presented results in this article, as well as on previously published results showed the high ecotoxicity of this compound, every precaution should be taken to prevent it getting into the environment.

## Figures and Tables

**Table 1:** Details of the spectrophotometric methods used for determining soil enzyme activity.

Enzyme	Buffer	Substrate	Incubation Temperature/Time	Wavelength	References
Carbon cycle					
DHA	0.1 M Tris buffer, pH 7.6	1% 2,3,5-triphenyltetrazolium chloride	25 °C/24 h	485 nm	[33]
LIP	100 mM NaH_2_PO_4_/NaOH buffer, pH 7.25	100 mM p-nitrophenyl butyrate	20 °C/10 min	400 nm	[34]
GLU	modified universal buffer *, pH 6.0	25 mM p-nitrophenyl-β-D-glucopyranoside	37 °C/1 h	400 nm	[35]
Phosphorus cycle					
ACP	modified universal buffer *, pH 6.5	115 mM p-nitrophenyl phosphate hexahydrate	37 °C/1 h	400 nm	[36]
ALP	modified universal buffer *, pH 11.0				
PD	0.05 M Tris buffer, pH 8.0	5 mM bis(p-nitrophenyl) phosphate	37 °C/1 h	400 nm	[37]
PT	modified universal buffer *, pH 10.0	23 mg tris(p-nitrophenyl) phosphate **	37 °C/1 h	400 nm	[38]
Nitrogen cycle					
URE	0.1 M borate buffer, pH 10.0	79.9 mM urea	37 °C/2 h	660 nm	[39]
NR	0.19 M ammonium chloride buffer, pH 8.5	25 mM KNO_3_	25 °C/24 h	520 nm	[40]
PROT	0.05 M Tris buffer. pH 8.1	2% casein	50 °C/2 h	700 nm	[41]
ArgD	water	11.5 M L-arginine	37 °C/3 h	630 nm	[42]

* The modified universal buffer was obtained by dissolving 12.1 g of tris(hydroxymethyl)aminomethane, 11.6 g of maleic acid, 14 g of citric acid monohydrate, and 6.3 g of boric acid in 500 mL of 1 M NaOH, and diluting the volume to 1000 cm^3^ with distilled water; ** insoluble in water; DHA dehydrogenases, CAT catalase, LIP lipase, GLU β-glucosidase, ACP acid phosphomonoesterase, ALP alkaline phosphomonoesterase, PD phosphodiesterase, PT phosphotriesterase, URE urease, NR nitrate reductase, PROT proteases, ArgD arginine deaminase.

**Table 2:** Activity of enzymes in the soil not exposed to tetrabutylphosphonium bromide [TBP][Br] in pot experiment with spring barley.

Enzyme	Time of Exposition (days)			
	1	7	14	21
Carbon cycle				
DHA (mg TPF kg^−1^ DM h^−1^)	1.65 ± 0.06 ^a^	1.62 ± 0.11 ^a^	1.64 ± 0.06 ^a^	1.61 ± 0.07 ^a^
CAT (mg H_2_O_2_ kg^−1^ DM h^−1^)	8.51 ± 0.31 ^a^	8.34 ± 0.25 ^a^	84.0 ± 0.31 ^a^	84.2 ± 0.20 ^a^
LIP (mg *p*-NP kg^−1^ DM h^−1^)	23.24 ± 1.06 ^a^	25.17 ± 1.87 ^a^	24.62 ± 0.92 ^a^	25.77 ± 1.39 ^a^
GLU (mg *p*-NP kg^−1^ DM h^−1^)	86.79 ± 1.97 ^a^	91.58 ± 6.04 ^a^	89.81 ± 1.53 ^a^	90.98 ± 0.97 ^a^
Phosphorus cycle				
ACP (mg *p*-NP kg^−1^ DM h^−1^)	75.60 ± 2.65 ^a^	75.56 ± 3.92 ^a^	77.14 ± 3.42 ^a^	77.68 ± 4.02 ^a^
ALP (mg *p*-NP kg^−1^ DM h^−1^)	128.27 ± 6.27 ^a^	129.95 ± 3.45 ^a^	134.48 ± 4.39 ^a^	129.47 ± 1.84 ^a^
PD (mg *p*-NP kg^−1^ DM h^−1^)	12.54 ± 0.86 ^a^	11.54 ± 0.54 ^a^	13.27 ± 1.28 ^a^	12.67 ± 1.20 ^a^
PT (mg *p*-NP kg^−1^ DM h^−1^)	8.59 ± 0.46 ^a^	9.30 ± 0.80 ^a^	8.53 ± 0.47 ^a^	8.80 ± 0.36 ^a^
Nitrogen cycle				
URE (mg N-NH_4_ kg^−1^ DM h^−1^)	73.68 ± 3.49 ^a^	74.75 ± 2.84 ^a^	77.09 ± 3.59 ^a^	75.92 ± 3.13 ^a^
NR (mg N-NO_2_^−^ kg^−1^ DM h^−1^)	2.59 ± 0.11 ^a^	2.56 ± 0.10 ^a^	2.64 ± 0.15 ^a^	2.64 ± 0.16 ^a^
PROT (mg Tyr kg^−1^ DM h^−1^)	26.37 ± 1.46 ^a^	26.86 ± 1.01 ^a^	26.06 ± 1.87 ^a^	26.19 ± 2.08 ^a^
ArgD (mg N-NH_4_ kg^−1^ DM h^−1^)	3.28 ± 0.21 ^a^	3.32 ± 0.10 ^a^	3.43 ± 0.19 ^a^	3.56 ± 0.13 ^a^

Data are expressed as mean ± SD; values denoted by the same letters in each line do not differ significantly on the level of *p* = 0.05 (Tukey HSD test); DHA—dehydrogenases, CAT—catalase, LIP—lipase, GLU—β-glucosidase, ACP—acid phosphomonoesterase, ALP—alkaline phosphomonoesterase, PD—phosphodiesterase, PT—phosphotriesterase, URE—urease, NR—nitrate reductase, PROT—proteases, ArgD—arginine deaminase, TPF—triphenylformazan, NP—nitrophenol, Tyr—tyrosine, DM—dry matter.

**Table 3:** Percentage share of factors contributing to enzyme activity in the soil exposed to tetrabutylphosphonium bromide [TBP][Br] in pot experiment with spring barley.

Variable Factors	Carbon Cycle				Phosphorus Cycle				Nitrogen Cycle			
	DHA	CAT	LIP	GLU	ACP	ALP	PD	PT	URE	NR	PROT	ArgD
D	67.64	78.63	87.23	15.02	62.58	74.30	35.43	18.54	64.10	81.81	73.86	85.77
ET	20.82	16.61	2.36	69.32	26.09	16.37	59.16	36.25	32.05	5.19	20.87	8.74
D × ET	7.67	3.97	8.97	9.89	6.78	8.62	4.40	10.15	3.37	6.08	4.94	4.68
Error	3.86	0.79	1.44	5.78	4.56	0.71	1.01	35.06	0.49	6.93	0.33	0.81

DHA—dehydrogenases, CAT—catalase, LIP—lipase, GLU—β-glucosidase, ACP—acid phosphomonoesterase, ALP—alkaline phosphomonoesterase, PD—phosphodiesterase, PT—phosphotriesterase, URE—urease, NR—nitrate reductase, PROT—proteases, ArgD—arginine deaminase, D—dose of [TBP][Br], ET—time of incubation.

**Table 4:** Integration of absorption peaks corresponding to the aliphatic and hydrophilic SOM fractions in the soil exposed to tetrabutylphosphonium bromide [TBP][Br] in pot experiment with spring barley.

Dose of [TBP][Br] (mg kg^−1^ DM)	A/C	B/C	A/B
Day 1			
0 (control)	0.516 ± 0.023 ^b^	3.696 ± 0.128 ^ab^	0.137 ± 0.009 ^h^
1	0.501 ± 0.019 ^b^	3.676 ± 0.110 ^abc^	0.134 ± 0.006 ^h^
10	0.508 ± 0.017 ^b^	3.825 ± 0.157 ^ab^	0.139 ± 0.004 ^gh^
100	0.478 ± 0.039 ^b^	3.777 ± 0.293 ^ab^	0.139 ± 0.005 ^gh^
1000	0.486 ± 0.034 ^b^	3.965 ± 0.164 ^a^	0.143 ± 0.005 ^fgh^
Day 7			
0 (control)	0.492 ± 0.092 ^b^	3.711 ± 0.218 ^abc^	0.139 ± 0.005 ^gh^
1	0.494 ± 0.016 ^b^	3.763 ± 0.357 ^ab^	0.135 ± 0.005 ^h^
10	0.543 ± 0.036 ^b^	3.384 ± 0.297 ^abcd^	0.161 ± 0.006 ^fgh^
100	0.658 ± 0.045 ^a^	3.170 ± 0.137 ^bcde^	0.208 ± 0.019 ^e^
1000	0.694 ± 0.013 ^a^	2.692 ± 0.142 ^de^	0.258 ± 0.015 ^bc^
Day 14			
0 (control)	0.491 ± 0.014 ^b^	3.639 ± 0.313 ^abc^	0.137 ± 0.003 ^h^
1	0.489 ± 0.030 ^b^	3.834 ± 0.308 ^ab^	0.137 ± 0.005 ^h^
10	0.547 ± 0.048 ^b^	3.248 ± 0.306 ^abcd^	0.169 ± 0.006 ^f^
100	0.686 ± 0.032 ^a^	3.001 ± 0.090 ^cde^	0.229 ± 0.017 ^de^
1000	0.694 ± 0.036 ^a^	2.456 ± 0.186 ^e^	0.283 ± 0.015 ^ab^
Day 21			
0 (control)	0.496 ± 0.015 ^b^	3.655 ± 0.349 ^abc^	0.137 ± 0.004 ^h^
1	0.500 ± 0.029 ^b^	3.869 ± 0.359 ^a^	0.136 ± 0.003 ^h^
10	0.546 ± 0.036 ^b^	3.257 ± 0.187 ^abcd^	0.168 ± 0.002 ^fg^
100	0.683 ± 0.009 ^a^	2.819 ± 0.107 ^de^	0.243 ± 0.007 ^cd^
1000	0.709 ± 0.020 ^a^	2.475 ± 0.178 ^e^	0.287 ± 0.015 ^a^

Data are expressed as mean ± SD; values denoted by the same letters in each column do not differ significantly on the level of *p* = 0.05 (Tukey HSD test); A peak height at 2932 cm^−1^ of Band A (2947–2858 cm^−1^); B peak height at 1631 cm^−1^ of Band B (1647–1633 cm^−1^); C peak height at 780 cm^−1^ of Band C (798–779 cm^−1^).

**Table 5:** Percentage share of factors contributing to the formation of aliphatic and hydrophilic SOM fractions in the soil exposed to tetrabutylphosphonium bromide [TBP][Br] in pot experiment with spring barley.

Variable Factor	A/C	B/C	A/B
D	52.48	39.10	61.48
ET	18.65	20.82	17.32
D × ET	22.06	23.35	19.28
Error	6.81	16.73	1.92

D dose of [TBP][Br], ET time of incubation; A peak height at 2932 cm^−1^ of Band A (2947–2858 cm^−1^); B peak height at 1631 cm^−1^ of Band B (1647–1633 cm^−1^); C peak height at 780 cm^−1^ of Band C (798–779 cm^−1^).

**Table 6:** Pearson correlation coefficients for the relationship between enzyme activity and organic matter characterization and ecotoxicity to *A. fischeri* of the soil samples exposed to tetrabutylphosphonium bromide [TBP][Br] in pot experiment with spring barley.

Enzyme	A/C	B/C	A/B	%T
Carbon cycle				
DHA	0.955 *	−0.902 *	0.934 *	0.684 *
CAT	−0.884 *	0.853 *	−0.911 *	−0.755 *
LIP	0.280	−0.122	0.151	0.068
GLU	−0.276	0.243	−0.259	−0.134
Phosphorus cycle				
ACP	0.097	0.022	−0.052	−0.152
ALP	−0.352	0.188	−0.212	−0.052
PD	−0.814 *	0.806 *	−0.851 *	−0.541 *
PT	−0.033	0.097	−0.091	−0.217
Nitrogen cycle				
URE	−0.823 *	0.828 *	−0.897 *	−0.790 *
NR	0.832 *	−0.839 *	0.907 *	0.879 *
PROT	0.843 *	−0.763 *	0.811 *	0.588 *
ArgD	−0.801 *	0.843 *	−0.894 *	−0.833 *

* significant at level of *p* = 0.05; DHA—dehydrogenases, CAT—catalase, LIP—lipase, GLU—β-glucosidase, ACP—acid phosphomonoesterase, ALP—alkaline phosphomonoesterase, PD—phosphodiesterase, PT—phosphotriesterase, URE—urease, NR—nitrate reductase, PROT—proteases, ArgD—arginine deaminase; A/C contribution of aliphatic components in SOM, B/C contribution of hydrophilic components in SOM, A/B abundance of hydrophobic and hydrophilic groups in SOM; %T percentage toxicity to *A. fischeri*.

## Data Availability

The data presented in this study are available on request from the corresponding author.
